# The complete mitochondrial genome of the snapping shrimp, *Alpheus brevicristatus* De Haan, 1844 (Crustacea, Decapoda, Alpheidae)

**DOI:** 10.1080/23802359.2025.2487072

**Published:** 2025-06-09

**Authors:** Yan-Bin Yang, Fang-Chao Zhu, Xin Liu, Lin-Tao Zhao, Shuo Yu, Xu-Yang Chen

**Affiliations:** a School of Resources, Environment and Materials, Guangxi University, Nanning, P. R. China; b Key Laboratory of Tropical Marine Ecosystem and Bioresource, Fourth Institute of Oceanography, Ministry of Natural Resources, Beihai, P. R. China; c Guangxi Key Laboratory of Beibu Gulf Marine Resources, Environment and Sustainable Development, Fourth Institute of Oceanography, Ministry of Natural Resources, Beihai, P. R. China; d Observation and Research Station of Coastal Wetland Ecosystem in Beibu Gulf, Ministry of Natural Resources, Beihai, P. R. China

**Keywords:** Mitochondrial genome, snapping shrimp, *Alpheus brevicristatus*, phylogenetic analysis

## Abstract

The snapping shrimp *Alpheus* is the genus with most species in the Alpheidae family. In this study, the mitochondrial genome of *Alpheus brevicristatus* De Haan, 1844 has been sequenced and analyzed. The circular mitogenome was 15,705 bp in length with an A+T content of 62.64%. It contained 37 genes typically found in metazoans, and one non-coding region. Phylogenetic analysis highly supported the placement of *A. brevicristatus* within Alpheidae. This study provides important mitochondrial genome data that could be used for further phylogenetic and evolutionary study of caridean snapping shrimps.

## 1. Introduction

The snapping shrimp genus *Alpheus* is the most diverse and abundant group within the Caridean shrimps, containing more than 300 species described to date (Sha et al. 2019). Most species of *Alpheus* are found in shallow tropical and subtropical marine waters, and typically burrow in soft sediment or live under rocks, shells and coral reefs (Zhong et al. 2019). Some species form intimate symbioses with other marine taxa, such as gobiid fishes (Hurt et al. 2009). However, due to its numerous morphological variations and the presence of cryptic taxa, the snapping shrimp has been considered as a particular challenge for taxonomist. Complete mitochondrial genome sequencing provides a powerful tool for the study of the evolutionary history and the genetic relationship of metazoans. *Alpheus brevicristatus* De Haan, 1844 has been found in almost all waters of China seas but has been rarely recorded or illustrated (Sha et al. 2019). In the present study, the complete mitochondrial genome sequence of *A. brevicristatus* has been reported and the authors believe that the mitogenome will provide valuable information for more accurate reconstruction of phylogenetic relationships.

## 2. Materials and methods

**Figure 1.**
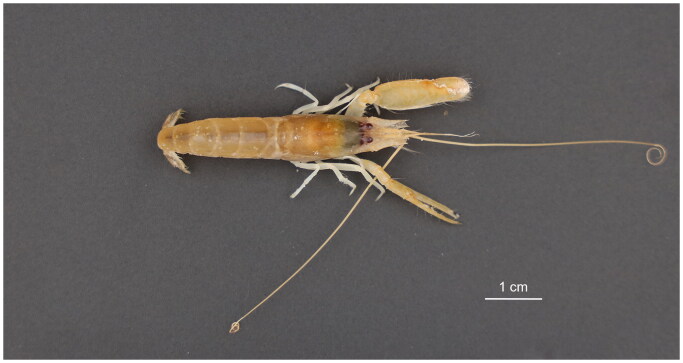
Specimen photograph for *Alpheus brevicristatus*. The photo was taken by Lin-Tao zhao.

The snapping shrimp specimen was collected from a shallow seagrass bed in Beihai, Guangxi province, China (21.4322° N, 109.2835° E) (Figure 1). The specimen was identified as *A. brevicristatus* based on its dactylus bearing plunger, compressed large chela and slender antennular peduncle. Moreover, its *cox1* sequence showed 97.8% similarity to the barcode record for *A. brevicristatus* in the BOLD identification system (Ratnasingham and Hebert 2007). The specimen was deposited at the Fourth Institute of Oceanography, Ministry of Natural Resources, P. R. China (Fang-Chao Zhu, zhufangchao@4io.org.cn) under the voucher number 2022040810.

Total genomic DNA was extracted from the intestine using a DNeasy PowerSoil Pro Kit (QIAGEN, Germany). Then, a genomic shotgun library was constructed with the NEBNext Ultra II DNA Library Prep Kit (NEB, USA), and sequenced on the Illumina NovaSeq 6000 platform (Personalbio Co., Shanghai, China). 32,386,777 pairs of raw reads (2×150 bp) were generated and subsequently trimmed using Trimmomatic v0.39 (Bolger et al. 2014). Adapters, low quality bases at both end of a read (below quality 3) and reads below the 50 bases long were removed. Mitochondrial genome was assembled using the NOVOPlasty v4.2 (Dierckxsens et al. 2017), in which the partial cytochrome oxidase subunit I gene of *A. brevicristatus* (GenBank accession number: HM180433) was used as seed sequence, and the complete mitochondrial genome of *A. inopinatus* (MG551491) was used as reference. The resulting circular sequence was annotated and verified with MITOS2 WebServer (https://usegalaxy.eu/) (Bernt et al. 2013). Boundaries of the PCGs and rRNAs were determined by alignment with the homologous genes of other amphipods. Organelle genome map was generated using the Proksee webserver (https://proksee.ca/) (Grant et al. 2023).

A maximum-likelihood (ML) phylogenetic tree was constructed based on 34 complete Caridean mitogenomes using PhyloSuite v1.2.3 (Zhang et al. 2020), and two Penaeidae shrimps were used as outgroups (Table S2). Thirteen mitochondrial protein-coding genes (PCGs) were extracted and aligned in batches using MAFFT v7.471 (Katoh and Standley 2013). After trimming with trimAl v1.2 (Capella-Gutiérrez et al. 2009), the aligned gene sets were concatenated. ModelFinder v2.2.0 (Kalyaanamoorthy et al. 2017) was used to select the best-fit partition model using BIC criterion. Finally, ML phylogenies were inferred using IQ-TREE v2.2.0 (Nguyen et al. 2015) under edge-linked partition model for 5,000 ultrafast bootstraps. The phylogenetic tree was visualized using the online tool iTOL v7 (https://itol.embl.de/) (Letunic and Bork 2007).

## 3. Results

**Figure 2.**
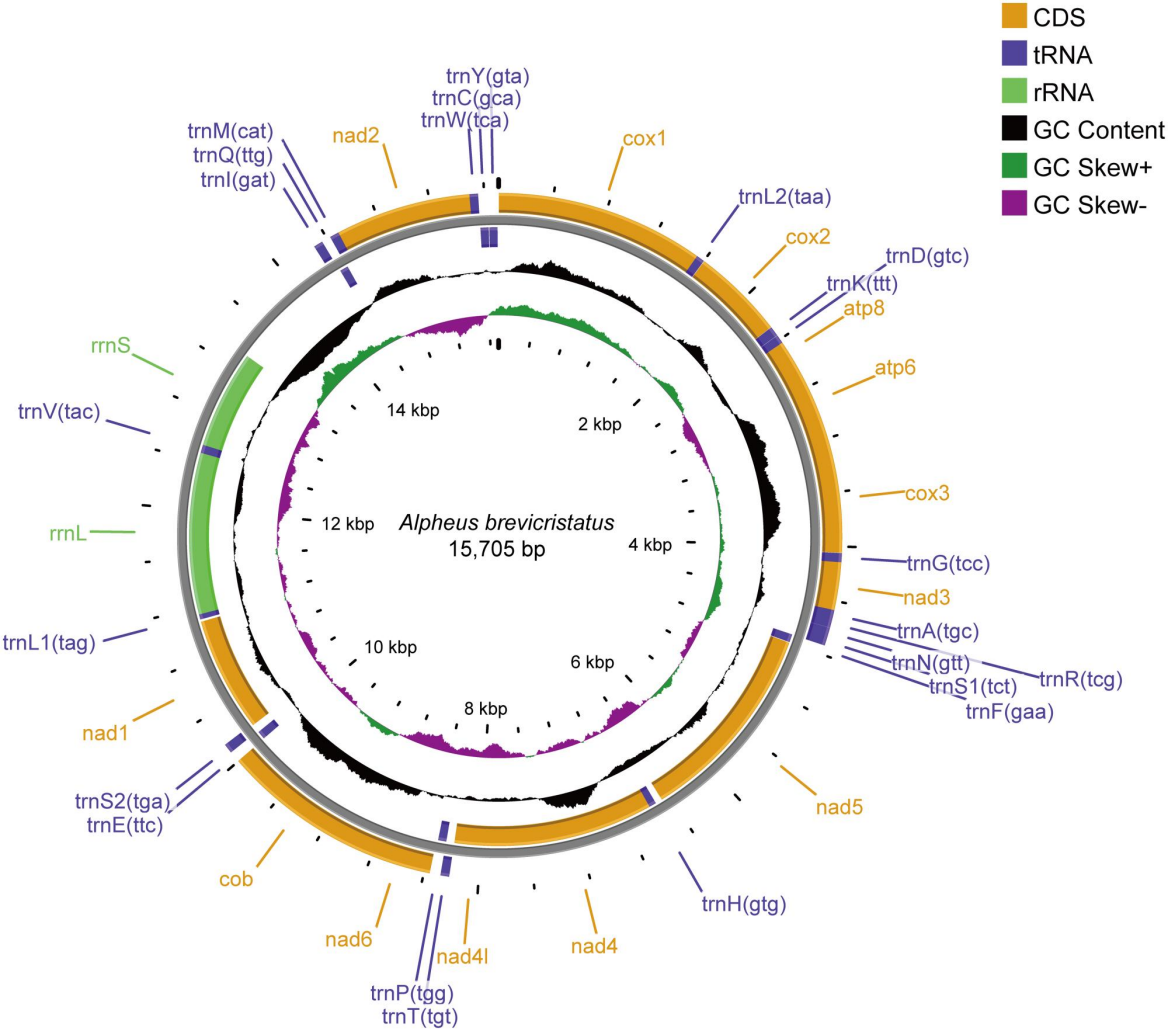
Mitochondrial genome structure of *Alpheus brevicristatus*. Protein coding, ribosomal RNA and transfer RNA genes are shown with abbreviations. The outer circle shows the gene arrangement and the genes in the inner are encoded on the light strand. The middle circle shows GC content, which the inner shows GC skew.

**Figure 3.**
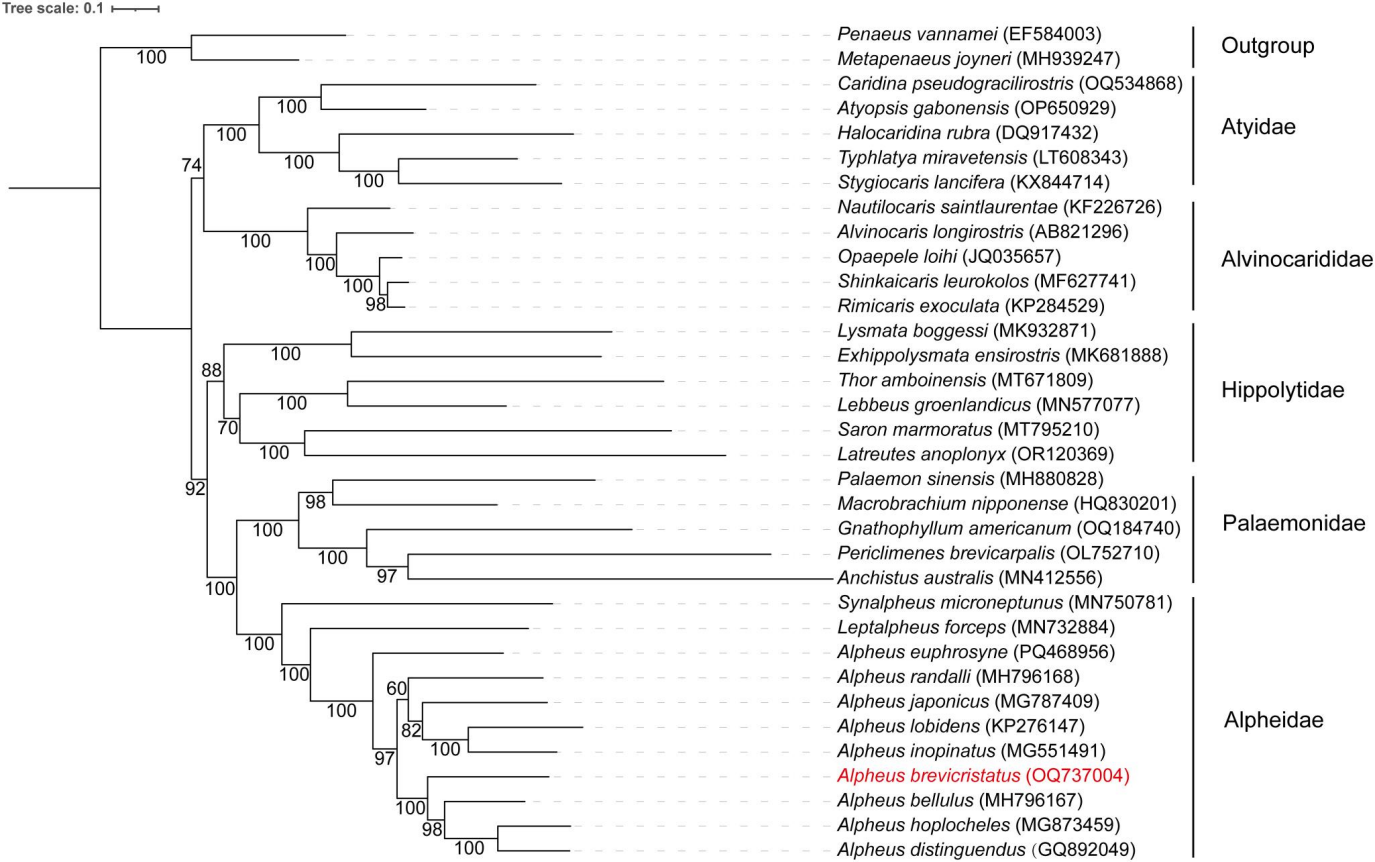
Maximum-likelihood phylogenetic tree based on sequences from 13 PCGs of the mitochondrial genome. Bootstrap values are indicated on the branches. All species in the tree are labeled with their scientific names and NCBI GenBank accession numbers on the right side. The position of *Alpheus brevicristatus* was highlighted in red.

The mitochondrial genome of *A. brevicristatus* was 15,705 bp in length (Figure 2), and the average read mapping depth was 253× (Figure S1). The overall base composition of the mitogenome was 33.22% A, 24.32% C, 13.05% G and 29.42% T, which showed a higher AT content (62.64%) than GC (37.36%). The mitogenome encoded the typical set of 37 metazoan genes, including 13 PCGs, 22 tRNA genes and 2 rRNA genes (Table S1). Only 1,114 bp non-coding nucleotides were found, with 159 bp in 17 intergenic regions and a 955 bp long non-coding region between the *rrnS* and *trnI* genes. Moreover, the entire *A. brevicristatus* mitogenome had 12 overlapping regions, each ranging in length from 1 to 23 bp. Nine PCGs and thirteen tRNAs were encoded on the heavy strand, while the remaining 15 genes were encoded on the light strand. Most of the PCGs had ATG or ATT as start codon except for *cox1* (TCG) and *atp8* (GTG). Twelve PCGs terminated the typical TAA or TAG as the stop codon, while only *nad3* ended with an incomplete T. The phylogenetic analysis revealed that *A. brevicristatus* formed a distinct clade with reported *Alpheus* species and was most closely related to *A. bellulus* (Figure 3).

## 4. Discussion and conclusion

In this study, the complete mitochondrial genome sequence of *A. brevicristatus* has been reported, and the genome structure and its phylogenetic relationship with other snapping shrimps have been investigated. The mitogenome of *A. brevicristatus* had a length of 15,705 bp and an AT content of 62.64%, which were close to the reported *Alpheus* mitogenomes (length ranged from 15,676 to 16,619 bp, and AT content ranged from 58.78 to 67.25%) (Qian et al. 2011; Zhong et al. 2019; Wang et al. 2020). Both *cox1* and *atp8* genes had an unusual start codon, and the non-canonical start codons TCG and GTG were previously observed in *A. distinguendus* and *A. japonicus* (Qian et al. 2011; Shen et al. 2012). It was reported that *cox1* was a hotspot for non-canonical start codons in invertebrate mitogenomes (Donath et al. 2019). Comparing with the ancestors of Caridea, the mitogenome gene order of *A. distinguendus* as well as most other *Alpheus* shrimps reported had the inversion and transposition of *trnE* (Wang et al. 2020). Besides, phylogenetic analysis highlighted a close relationship between *A. bellulus* and *A. brevicristatus*. The results of our study could expand the mitochondrial genome data of the Alpheidae family, and will provide useful genetic information for their phylogenetic and taxonomic classification.

## Supplementary Material

Supplemental Material

## Data Availability

The genome sequence data that support the findings of this study are openly available in GenBank of NCBI at [https://www.ncbi.nlm.nih.gov] under the accession no. OQ737004. The associated BioProject, BioSample and SRA numbers are PRJNA1210165, SAMN46240869, and SRR31990051, respectively. The raw data of high-throughput sequencing have also been deposited in the China National GeneBank Database with accession number CNX1160935.
