# Supplementary material for: The complete mitochondrial genome of the snapping shrimp, Alpheus brevicristatus De Haan, 1844 (Crustacea, Decapoda, Alpheidae)
Source: Mitochondrial DNA B Resour. 2025 Jun 9;10(7):554–7. doi: 10.1080/23802359.2025.2487072 (PMC12152993; doi:10.1080/23802359.2025.2487072)
Supplement: Supplemental Material [file TMDN_A_2487072_SM1381.docx]

Table S1. The organization of the mitochondrial genome of *Alpheus brevicristatus*.

| Gene | Location | | Codon |  | Length (bp) | Strand | Intergenic nucleotides^a^ |
| --- | --- | --- | --- | --- | --- | --- | --- |
|  | From | To | Start | Stop |  |  |  |
| *cox1* | 1 | 1539 | TCG | TAA | 1539 | + | -5 |
| *trnL2* | 1535 | 1600 |  |  | 66 | + | 4 |
| *cox2* | 1605 | 2288 | ATG | TAA | 684 | + | 3 |
| *trnK* | 2292 | 2362 |  |  | 71 | + | 3 |
| *trnD* | 2366 | 2427 |  |  | 62 | + | 2 |
| *atp8* | 2430 | 2588 | GTG | TAA | 159 | + | -7 |
| *atp6* | 2582 | 3256 | ATG | TAA | 675 | + | -1 |
| *cox3* | 3256 | 4044 | ATG | TAG | 789 | + | 3 |
| *trnG* | 4048 | 4113 |  |  | 66 | + | 0 |
| *nad3* | 4114 | 4465 | ATT | T(AA) | 352 | + | 0 |
| *trnA* | 4466 | 4531 |  |  | 66 | + | 0 |
| *trnR* | 4532 | 4596 |  |  | 65 | + | 2 |
| *trnN* | 4599 | 4665 |  |  | 67 | + | 0 |
| *trnS1* | 4666 | 4732 |  |  | 67 | + | -1 |
| *trnF* | 4732 | 4796 |  |  | 65 | - | 6 |
| *nad5* | 4803 | 6464 | ATT | TAG | 1662 | - | 57 |
| *trnH* | 6522 | 6585 |  |  | 64 | - | -5 |
| *nad4* | 6581 | 7918 | ATG | TAA | 1338 | - | -7 |
| *nad4L* | 7912 | 8211 | ATG | TAA | 300 | - | 3 |
| *trnT* | 8215 | 8278 |  |  | 64 | + | 0 |
| *trnP* | 8279 | 8344 |  |  | 66 | - | 14 |
| *nad6* | 8359 | 8865 | ATT | TAA | 507 | + | -1 |
| *cob* | 8865 | 10001 | ATG | TAA | 1137 | + | 4 |
| *trnE* | 10006 | 10071 |  |  | 66 | - | 12 |
| *trnS2* | 10084 | 10153 |  |  | 70 | + | -10 |
| *nad1* | 10144 | 11082 | ATG | TAA | 939 | - | 20 |
| *trnL1* | 11103 | 11167 |  |  | 65 | - | -23 |
| *rrnL* | 11145 | 12477 |  |  | 1333 | - | -11 |
| *trnV* | 12467 | 12533 |  |  | 67 | - | 0 |
| *rrnS* | 12534 | 13336 |  |  | 803 | - | 71 |
| OH | 13408 | 13957 |  |  | 550 | + | 12 |
| OL | 13970 | 14006 |  |  | 37 | - | 285 |
| *trnI* | 14292 | 14358 |  |  | 67 | + | 6 |
| *trnQ* | 14365 | 14431 |  |  | 67 | - | -5 |
| *trnM* | 14427 | 14493 |  |  | 67 | + | 0 |
| *nad2* | 14494 | 15492 | ATT | TAA | 999 | + | -2 |
| *trnW* | 15491 | 15556 |  |  | 66 | + | 3 |
| *trnC* | 15560 | 15625 |  |  | 66 | - | 6 |
| *trnY* | 15632 | 15697 |  |  | 66 | - | 11 |

a: the positive number indicates interval base pairs between genes, while the negative number indicates the overlapping base pairs between genes.

Table S2. The shrimp mitochondrial genomes used for phylogenetic inference.

| Species | Accession number | Reference |
| --- | --- | --- |
| Penaeus vannamei | EF584003 | Unpublished |
| Metapenaeus joyneri | MH939247 | Meng et al., 2019 |
| Caridina pseudogracilirostris | OQ534868 | Soundharapandiyan et al., 2023 |
| Atyopsis gabonensis | OP650929 | Sun et al., 2024 |
| Halocaridina rubra | DQ917432 | Ivey & Santos, 2007 |
| Typhlatya miravetensis | LT608343 | Jurado-Rivera et al., 2016 |
| Stygiocaris lancifera | KX844714 | Unpublished |
| Nautilocaris saintlaurentae | KF226726 | Kim et al., 2013 |
| Alvinocaris longirostris | AB821296 | Unpublished |
| Opaepele loihi | JQ035657 | Unpublished |
| Shinkaicaris leurokolos | MF627741 | Sun et al., 2018 |
| Rimicaris exoculata | KP284529 | Yu et al., 2016 |
| Lysmata boggessi | MK932871 | Unpublished |
| Exhippolysmata ensirostris | MK681888 | Ye et al., 2021 |
| Thor amboinensis | MT671809 | Wang et al., 2020b |
| Lebbeus groenlandicus | MN577077 | Kim et al., 2019 |
| Saron marmoratus | MT795210 | Wang et al., 2021 |
| Latreutes anoplonyx | OR120369 | Sun et al., 2024 |
| Palaemon sinensis | MH880828 | Zhao et al., 2019 |
| Macrobrachium nipponense | HQ830201 | Unpublished |
| Gnathophyllum americanum | OQ184740 | Sung et al., 2023 |
| Periclimenes brevicarpalis | OL752710 | Peng et al., 2024 |
| Anchistus australis | MN412556 | Liu, 2019 |
| Synalpheus microneptunus | MN750781 | Chak et al., 2020 |
| Leptalpheus forceps | MN732884 | Unpublished |
| Alpheus euphrosyne | PQ468956 | Unpublished |
| Alpheus randalli | MH796168 | Wang et al., 2020a |
| Alpheus japonicus | MG787409 | Unpublished |
| Alpheus lobidens | KP276147 | Unpublished |
| Alpheus inopinatus | MG551491 | Unpublished |
| ***Alpheus brevicristatus*** | **OQ737004** | **This study** |
| Alpheus bellulus | MH796167 | Wang et al., 2020a |
| Alpheus hoplocheles | MG873459 | Zhong et al., 2019 |
| Alpheus distinguendus | GQ892049 | Qian et al., 2011 |

Figure S1. The estimated coverage depths from Illumina sequencing reads for *Alpheus brevicristatus* mitogenome.


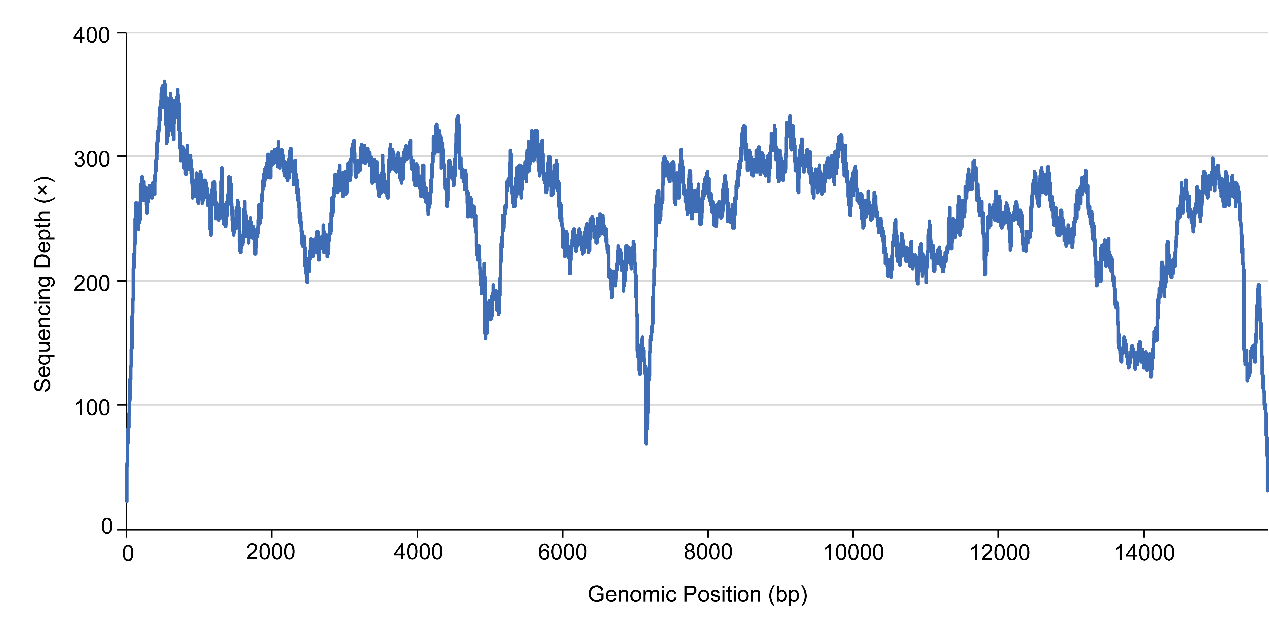


**Reference**

Chak ST, Barden P, Baeza JA. 2020. The complete mitochondrial genome of the eusocial sponge-dwelling snapping shrimp *Synalpheus microneptunus*. Scientific Reports. 10: 7744.

Ivey JL & Santos SR. 2007. The complete mitochondrial genome of the Hawaiian anchialine shrimp *Halocaridina rubra* Holthuis, 1963 (Crustacea: Decapoda: Atyidae). Gene. 394(1-2): 35-44.

Jurado-Rivera JA, Jaume D, Juan C, Pons J. 2016. The complete mitochondrial genome of the cave shrimp *Typhlatya miravetensis* (Decapoda, Atyidae) and its systematic position. Mitochondrial DNA Part B. 1(1): 847-848.

Kim J, Choi JP, Kim HS, Jo Y, Min WG, Yum S, Bhak J. 2019. The complete mitochondrial genome of a Dokdo shrimp, *Lebbeus groenlandicus*. Mitochondrial DNA Part B. 4(2): 4196-4197.

Kim SJ, Pak SJ, Ju SJ. 2013. Mitochondrial genome of the hydrothermal vent shrimp *Nautilocaris saintlaurentae* (Crustacea: Caridea: Alvinocarididae). Mitochondrial DNA. 26(1): 127-128.

Liu H. 2019. Sequence and phylogenetic analysis of the mitochondrial genome for a giant clam commensal shrimp *Anchistus australis* (Decapoda: Caridea: Palaemonidae). Mitochondrial DNA Part B. 5(1): 312-313.

Meng X, Gao B, Liu P. 2019. The complete mitochondrial genome of the Shiba shrimp *Metapenaeus joyneri* (Miers, 1880) (Decapoda: Penaeidae). Mitochondrial DNA Part B. 4(1): 465-467.

Peng C, Fan S, Peng PF. 2024. Characterization of complete mitochondrial genome of *Periclimenes brevicarpalis* (Decapoda: Palaemonidae). Mitochondrial DNA Part B. 9(10): 1414-1417.

Soundharapandiyan N, Rajaretinam RK, Wilson Alphonse CR. 2023. Exploring the mitochondrial genome of *Caridina pseudogracilirostris*: a comparative analysis within the Atyidae Family. Molecular Biology Reports. 50: 8121-8131.

Sun Y, Liu W, Chen J, Li J, Ye Y, Xu K. 2024. Sequence comparison of the mitochondrial genomes of five caridean shrimps of the infraorder Caridea: phylogenetic implications and divergence time estimation. BMC Genomics. 25: 968.

Sung CH, Cheng CC, Huang CW, Wang LJ. 2023. Characterization and phylogenetic analysis of the first complete mitochondrial genome of *Gnathophyllum americanum* (Guérin-Méneville, 1855) (Malacostraca: Decapoda: Palaemonidae). Fishes. 8(5): 238.

Wang Y, Zeng L, Wen J, Li X, Huang Y, Sun Y, Zhao J. 2020b. The complete mitochondrial genome of *Thor amboinensis* (Hippolytidae, Decapoda). Mitochondrial DNA Part B. 5(3): 3077-3078.

Wang Y, Zeng L, Wen J, Li X, Huang Y, Sun Y, Zhao J. 2021. Characterization of the complete mitochondrial genome of *Saron marmoratus* (Hippolytidae, Decapoda) and its phylogenetic analysis. Mitochondrial DNA Part B. 6(1): 124-126.

Ye Y, Miao J, Guo Y, Gong L, Jiang L, Lü Z, Xu K, Guo B. 2021. The first mitochondrial genome of the genus *Exhippolysmata* (Decapoda: Caridea: Lysmatidae), with gene rearrangements and phylogenetic associations in Caridea. Scientific Reports. 11: 14446.

Yu YQ, Liu LX, Li HW, Lu B, Fan YP, Yang JS. 2016. The complete mitogenome of the Atlantic hydrothermal vent shrimp *Rimicaris exoculata* Williams & Rona 1986 (Crustacea: Decapoda: Alvinocarididae). Mitochondrial DNA Part A. 27(5): 3115-3117.

Zhao Y, Zhu X, Li Y, Han Z, Xu W, Dong J, Wei H, Li X. 2019. Mitochondrial genome of Chinese grass shrimp, *Palaemonetes sinensis* and comparison with other Palaemoninae species. Scientific Reports. 9: 17301.
